# The Transgenerational Link: Breeder Gut Microbiota and Broiler Progeny Development

**DOI:** 10.1002/mbo3.70174

**Published:** 2025-11-27

**Authors:** Gladys Maria Pangga, Stephen Bamford, Anne Richmond, Nicolae Corcionivoschi, Umer Zeeshan Ijaz, Ozan Gundogdu

**Affiliations:** ^1^ London School of Hygiene & Tropical Medicine London UK; ^2^ Pilgrim's Europe Ltd. Craigavon UK; ^3^ Agri‐Food Biosciences Institute Belfast UK; ^4^ University of Glasgow Glasgow UK

**Keywords:** breeder, broilers, microbial transfer, microbiota, parent, poultry, progeny

## Abstract

The gut microbiome of breeder hens plays a pivotal role in reproductive efficiency, egg quality, and progeny development. Its composition is shaped by host factors such as age and genetics, as well as environmental influences, including diet and management practices. Importantly, the breeder gut microbiome is not only dynamic but also responsive to targeted interventions that can enhance intestinal health, metabolic function, and laying performance. Vertical transmission of maternal microbes through the cloaca and egg components provides offspring with a foundational microbial community, with the yolk sac serving as a critical reservoir for early colonisers that influence gut maturation, immunity, and growth. Emerging evidence further demonstrates that maternal nutritional strategies can programme the gut microbiota of progeny and intestinal development, highlighting the breeder microbiome as both a determinant and mediator of transgenerational performance. These insights underscore the potential of microbiome‐focused approaches to improve reproductive success and sustainability in poultry production.

## Graphical abstract

The breeder hen gut microbiome, shaped by maternal factors and targeted nutrition, drives reproductive performance and transmits beneficial microbes that programme gut health, immunity, and growth in progeny. Microbiome‐focused interventions can optimise egg quality, hatchability, progeny performance, and sustainability in poultry production.

## 1 Introduction

The gastrointestinal (GIT) microbiome is a fundamental component of the chicken host, underpinning numerous aspects of poultry health and productivity (Diaz Carrasco et al. 2019). This complex and dynamic microbial community performs essential roles in nutrient metabolism, immune system maturation, and protection against pathogen colonisation (Clavijo and Flórez 2018). Preservation of microbiota homoeostasis is therefore critical; however, it is inherently influenced by host genetics, physiology, and environmental factors (Diaz Carrasco et al. 2019). While its importance is well‐established in broilers and laying hens, the gut microbiome of breeder hens, despite their foundational role in reproduction and transgenerational microbial transfer (Lee et al. 2019), remains underexplored.

Modern poultry production operates within a highly structured, vertically integrated system designed to maximise efficiency and performance (Dittoe et al. 2019; Siegel 2014). At its foundation are broiler breeder hens and roosters, genetically selected parent stock whose primary role is to produce fertilised eggs (Jong and Emous 2017). While these breeders are not primarily intended for meat production, they serve as the reproductive cornerstone of the broiler industry and are maintained under tightly controlled nutritional and environmental conditions to support optimal egg production and fertility (Jong and Emous 2017). Once laid, eggs are disinfected and incubated in hatcheries, after which newly hatched chicks are transferred to grow‐out facilities for meat production, referred to as broilers. These broiler chickens are intensively selected for rapid growth, feed efficiency, and high meat yield, typically reaching market weight within 5–7 weeks (Siegel 2014; Baxter et al. 2021).

Although genetics, nutrition, and husbandry practices have traditionally been viewed as the primary drivers of broiler performance (Dittoe et al. 2019), increasing attention has turned toward maternal influences, particularly the role of the breeder gut microbiota in shaping progeny outcomes. Emerging evidence indicates that vertical transmission of microbial communities from the hen to the chick plays a pivotal role in establishing the early‐life microbiome of broiler chickens (Ding et al. 2017; Gao et al. 2025). These maternal microbial inputs have been shown to influence progeny immune function, gut development, and growth performance (Gao et al. 2025). It has also been established that various maternal characteristics, such as age and nutrition, affect progeny outcomes, including embryo villi development, weight and hatchability (Chang et al. 2016; Machado et al. 2020). Consequently, there is growing interest in targeted maternal interventions, including dietary supplementation with probiotics, prebiotics, and phytochemicals to modulate breeder gut microbiota in ways that confer transgenerational benefits (Gao et al. 2023; Wang et al. 2021a; Zhen et al. 2021).

Given the importance of gut microbiome in poultry health and performance and accumulating evidence of the link between the breeder parent and its progeny, maintaining a beneficial gut microbial environment in breeders is therefore essential not only for sustaining their own reproductive performance but also for optimising the development and productivity of their progeny. This review consolidates current knowledge of the breeder gut microbiome, its dynamic nature across the production cycle, and its mechanistic links with broiler progeny performance, with a particular emphasis on the transgenerational impact of microbiota‐mediated maternal effects and the potential of microbiome‐targeted interventions in broiler breeder management.

## 2 Development of the Breeder Gut Microbiome

The general chicken microbiome has been characterised extensively in previous reviews (Diaz Carrasco et al. 2019; Clavijo and Flórez 2018; Chica Cardenas et al. 2021; Fathima et al. 2022). In brief, the development of gut microbiota is a dynamic process that begins shortly after hatching, as the GIT is typically sterile at hatch (Fathima et al. 2022). In modern commercial hatchery settings, chicks acquire their microbiota predominantly from environmental sources rather than through direct hen contact, which was the historical method of vertical transmission (Fathima et al. 2022; Aruwa et al. 2021). This colonisation of the gut involves a succession of microbial populations, starting with facultative aerobes, such as coliforms and faecal Streptococcus, which become abundant by day 3 post‐hatch (Fathima et al. 2022; Aruwa et al. 2021). Their growth and oxygen consumption create reducing conditions that promote the subsequent colonisation by obligate anaerobes. While the small intestinal microbiota is generally established by around 2 weeks, the caecal microbiota becomes fully established by 6‐7 weeks of age, demonstrating an increasing diversity over time (Fathima et al. 2022).

**Figure 1:**
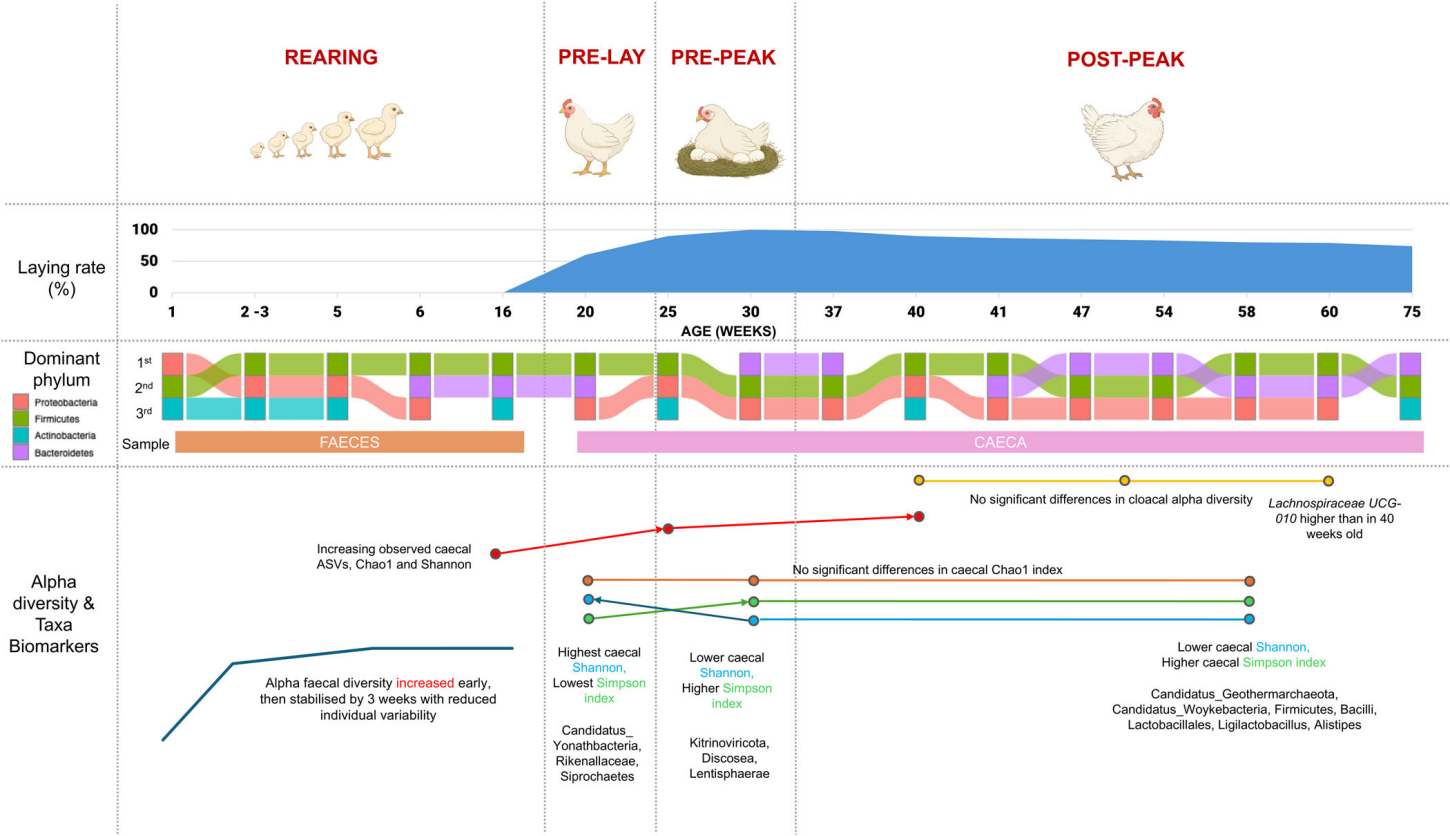
Summary of gut microbial shifts during development and production phases. Based on (Wang et al. 2021a; Díaz‐Sánchez et al. 2019; Amevor et al. 2022; Feng et al. 2021; Prentza et al. 2022; Robinson et al. 2003; Shi et al. 2024; Shterzer et al. 2020; Van Syoc et al. 2022; Xiao et al. 2023; Yang et al. 2020).

Specifically, in pedigree broiler breeders, the faecal microbiome is dynamic in early life but stabilises after 3 weeks of age, independent of genetic lineage (Díaz‐Sánchez et al. 2019). Time‐series experiments show that at hatching, the faecal microbial communities are dominated by the phylum Proteobacteria (Díaz‐Sánchez et al. 2019). As development progresses, a shift occurs, leading to a community dominated by Firmicutes, with increasing abundances of Actinobacteria and Bacteroidetes (Díaz‐Sánchez et al. 2019). In addition, the majority of operational taxonomic units (OTUs) in the gut microbiome persist over host development, becoming common and stable across chickens of the same genetic lineage after 3 weeks of age (Díaz‐Sánchez et al. 2019) (Figure 1).

**Figure 2:**
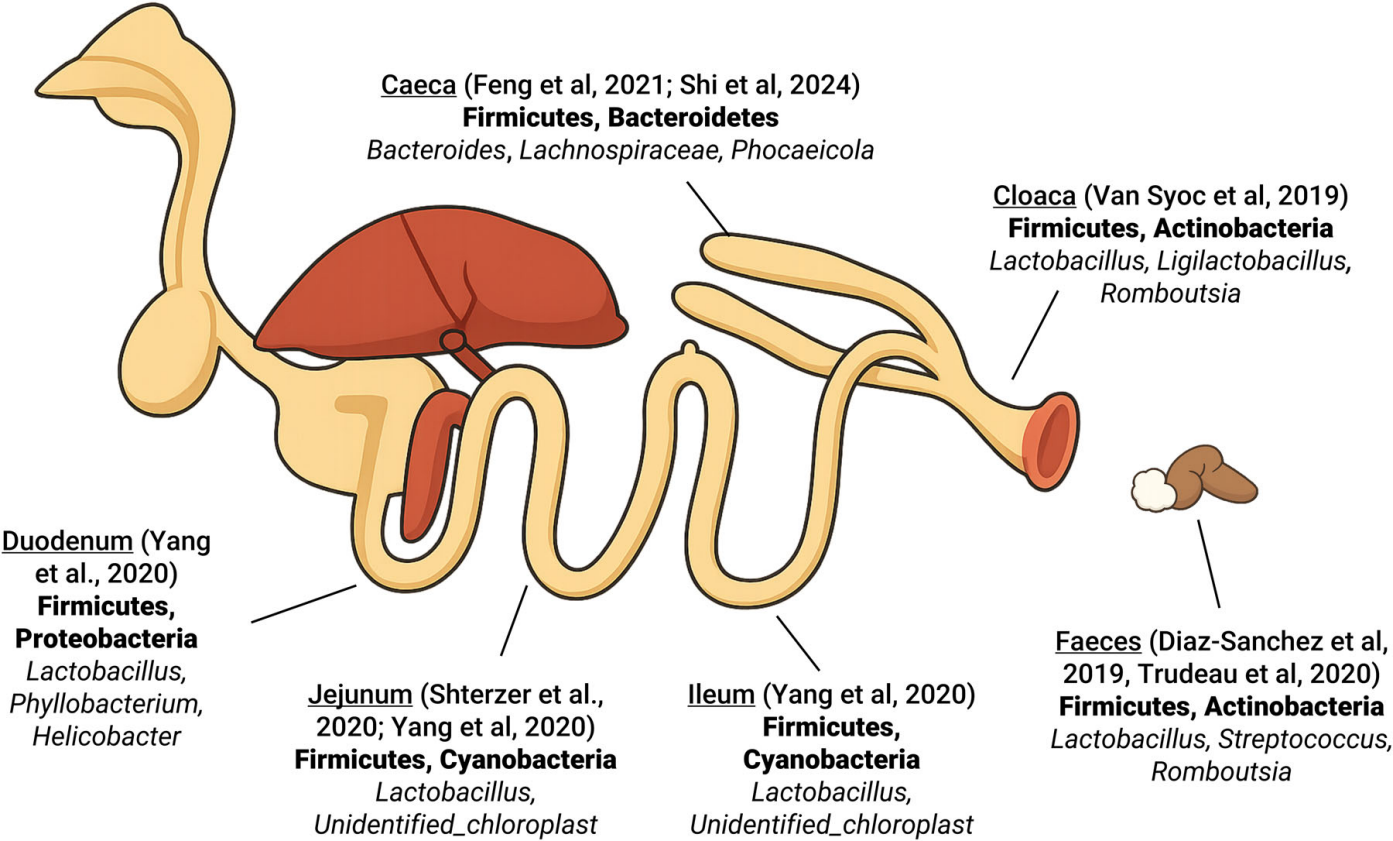
Regional abundance and representative taxa of broiler gut microbiota. Based on Diaz‐Sanchez et al. 2019; Feng et al. 2021; Shterzer et al. 2020; Shi et al. 2024; Van Syoc et al. 2022; Yang et al. 2020; Trudeau et al. 2020.

As chickens mature, their gut microbiome becomes more diverse and undergoes compositional shifts. Each region of the GIT supports a distinct microbial community, reflecting its specific digestive functions. Overall, the chicken gut is estimated to contain over 915 bacterial species, spanning 117 genera and 13 phyla (Wei et al. 2013). The dominant bacterial phyla generally include Firmicutes, Bacteroidetes, and Proteobacteria, with genera such as *Lactobacillus*, *Ruminococcus*, *Clostridium*, and *Bacteroides* becoming prevalent, particularly in the caeca, which typically exhibits the highest microbial density and diversity (Clavijo and Flórez 2018; Chica Cardenas et al. 2021). A more recent study of breeder broilers identified an even broader microbial landscape in the caeca, reporting a total of 231 phyla, 440 classes, 785 orders, 1490 families, 4862 genera, and 24,908 species (Shi et al. 2024). Among these, the three most abundant phyla remained Firmicutes, Bacteroidota, and Proteobacteria, while the dominant genera were *Bacteroides*, *Phocaeicola*, *Parabacteroides*, and *Alistipes* (Shi et al. 2024). Other gut samples used for microbial profiling of breeder hens include cloacal swabs, and contents of the jejunum, ileum and duodenum; Phylum dominance discrepancies can be observed among types, which is consistent with regional differences in the gut microbiome of broilers (Feye et al. 2020). (Figure 2).

## 3 Factors Affecting Breeder Hen Gut Microbiome

The composition and function of the breeder hen microbiome are influenced by a multitude of factors, contributing to its dynamic nature:

### 3.1 Age and Production Phase

The gut microbiome develops rapidly during early life but continues to fluctuate as breeder hens progress through distinct reproductive stages, each with specific metabolic and physiological demands (Jong and Emous 2017; Shi et al. 2024). A typical breeder hen production phase is mainly divided into three reproductive stages: Pre‐lay (20–24 weeks): Photo‐stimulation initiates sexual maturity; pre‐peak (25–32 weeks): Marks the transition from sexual maturity to peak egg production; post‐peak (33–50/55 weeks): Characterised by a gradual decline in production. Post‐peak can also include a late/decline stage around 55–75 weeks old). Each stage involves specific physiological and metabolic demands, emphasising the importance of disease prevention and consistent health management throughout the bird's life cycle (Jong and Emous 2017; Robinson et al. 2003).

These changing demands also influence the gut microbial diversity and composition, which is dynamic across the production cycle (Shi et al. 2024). Around peak production (~30 weeks), microbial abundance peaks while diversity declines, coinciding with shifts in hormone and amino acid metabolism and the appearance of stage‐specific microbial biomarkers (Shi et al. 2024). Similarly, Prentza et al. (2022) reported significant age‐related changes in caecal microbiota composition and diversity were reported at 15, 25, and 40 weeks (Prentza et al. 2022). Microbial communities clustered distinctly by age, with a total of 15,582 amplicon sequence variants (ASVs) observed. The authors also observed a pronounced increase in alpha diversity between 15 and 25 weeks, whereas the increase was comparatively attenuated between 25 and 40 weeks (Prentza et al. 2022).

Additional studies have examined microbiota changes during post‐peak stages, spanning ages from 37 to 75 weeks, further highlighting the complexity and variability of the breeder gut ecosystem over time (Gao et al. 2025; Amevor et al. 2022; Feng et al. 2021; Shterzer et al. 2020) (Figure 1). Trends on breeder microbial changes, including dominance of proteobacteria and of firmicutes in early age (Díaz‐Sánchez et al. 2019) and increasing microbial diversity from rearing to peak‐laying stages (Prentza et al. 2022; Shi et al. 2024), and dominance of Bacteroidetes during peak laying phases and old age (Wang et al. 2021a; Amevor et al. 2022; Shi et al. 2024; Shterzer et al. 2020), are similarly observed in layer chickens (Joat et al. 2023; Xu et al. 2023).

While these findings highlight broad patterns, discrepancies remain in the relative timing and taxonomic resolution of reported shifts, likely reflecting differences in diet, environment, and sequencing depth. Importantly, most studies remain cross‐sectional, limiting causal inference about whether microbial changes drive or simply reflect physiological transitions. Multi‐omics studies are beginning to bridge this gap by linking microbial shifts to metabolic and hormonal pathways (Shi et al. 2024), but more strain‐level and functional analyses are needed to clarify the mechanistic role of the microbiome across the breeder production cycle.

### 3.2 Host Genetics

As with broilers, the breed type or genetics of a bird can influence its microbiome. A study by Díaz‐Sánchez et al. (2019) reported distinct differences between two breeder pedigree lines (Line A and Line B), particularly in the abundance of the phylum Bacteroidetes during later developmental stages (6 and 16 weeks of age) (Díaz‐Sánchez et al. 2019). Meanwhile, Shterzer et al. (2024) reported marked differences in the caecal bacterial communities when comparing a modern commercial breeding dam line (Cobb) with a legacy broiler line, explicitly concluding that genetic changes resulting from breeding programmes led to alterations in the gut bacterial community (Shterzer et al. 2024). A key distinction was the high abundance of the *Akkermansia* genus in Cobb dams, which was largely absent in Legacy dams, suggesting a potential association between this bacterial group and host phenotypes achieved through genetic selection for traits like fast growth and high meat yield.

### 3.3 Diet and Nutrition Interventions

Diet is one of the most powerful levers for shaping the gut microbiome, and in breeder hens, it has direct implications for reproductive efficiency and egg quality (Amevor et al. 2022). A growing body of work demonstrates that targeted nutritional strategies can alter microbial composition and activity, yet the evidence remains uneven and often descriptive (Table 1).

**Table 1:** Overview of breeder chicken gut health interventions and corresponding effects on microbiome and performance.

Supplement	Dose	Breeder age	Sample	Diversity metrics	Taxa correlated	Functions/metabolites	Hen production traits	Egg quality and hatching performance	Other important remarks	Source
Phytochemicals and plant‐bioactive substances										
Chinese herbal mixture	1 g/kg	30–40	Caecal	↓ Shannon, ↓ Simpson	↓ Proteobacteria, ↓ Desulfovibrio, ↓ Staphylococcus, ↓ Pseudomonas, ↑ Bacteroidetes, ↑ Spirochaetes,	Not determined	↑ ELR, ↓ FCR	↓ Embryo mortality, ↑ hatching of fertile eggs, ↑ offspring weight	Desulfovibrio and Proteobacteria: (‐) corr. w/ELR, IL‐6; Bacteroidetes: (+) corr. w/ELR, IgG	Liu et al. 2023
Quercetin	0.4 g/kg	65–75	Caecal	No change	↑ Bacteroidaceae, ↑ Desulfovibrionaceae, ↑ Peptotostretococcaceae, ↑ Fusobacteriaceae	↑ DL‐Carnitine, ↑ Cuminaldehyde, ↑ ubiquinone, ↑ terpenoid–quinone biosynthesis, ↑ regulation of actin cytoskeleton, ↑ insulin secretion, ↑ pancreatic secretion, ↑ nicotine addiction	No change	Not determined	Oribacterium: (‐) corr. w/Aflatoxin M1	Amevor et al. 2022
Combination of quercetin and vitamin E	0.4 g/kg and 0.2 g/kg	67–75	Caecal	No change	↑ Bifidobacteriaceae, ↑ Lachnospiraceae, ↑ Tannerellaceae, ↑ Mathonobacteriaceae, ↑ Barnesiellaceae, ↑ Prevotellaceae	↑ DL‐Carnitine, ↑ Cuminaldehyde, ↑ Phylloquinone, ↑ Succinoylpyridine, ↑ 1‐Naphthol, ↓ acetylcholine |, ↑ cP450 metabolism of xenobiotics	↑ ADFI, ↑ BW	Not determined	(S)‐equol (‐) corr. w/*Alistipes* and *Chlamydia*	Amevor et al. 2022
Soyasaponin	200 mg/kg	NA	Jejunal/Ileal, cloaca	Not determined	↑ Rhodococcus, ↑ Enterococcus, ↑ Bacteroides, ↓ Pseudomonas	Not determined	Not determined	Not determined	↑ microbial overlap between magnum and intestines; ↑ E2, FSH, and LH	Gao et al. 2025
Grape seed extracts	1%–2%	4–40 (1%), 38–40 (2%)	Caecal	No change	↑ Bifidobacteriaceae, ↑ Lactobacillaceae, ↑ Lachnospiraceae, ↑ Succinivibrionaceae, ↓ Rikenellaceae, ↓ Tannerellaceae	Not determined	↓ BW (2%)	↑ shell strength, ↑ EW	↓ fatness, ↓ plasma lipid and phenolic metabolites, ↓ caecal anti‐inflammatory markers (2%)	Grandhaye et al. 2020
Microencapsulated blend (AviPlus)	500 g/MT	0–3	Jejunal/Ileal	No change	↑ Lactobacillaceae, ↑ Lachnospiraceae, ↑ Peptostreptococcaceae, ↑ Enterobacteriaceae, ↑ Clostridiaceae	Not determined	Not determined	Not determined	Supplement supported response against necrotic enteritis	Dittoe et al. 2023
Probiotics and microbial metabolites										
Monobutyrin	250 mg/kg	33–41	Caecal	No change	No correlation found	No correlation found	No differences	↑ EW, ↓ egg breaking rate	↑ serum total protein	Feng et al. 2021
Yeast β‐glucan	200 mg/kg	43–51	Ileal	↑ Shannon, ↓ Simpson index	↑ Lactobacillus, ↑ Bacilli, ↑ Lactobacillales, ↑ Lactobacillaceae, ↑ Enterobacteriales, ↑ Enterobacteriaceae, ↑ *Escherichia‐Shigella*	↑ antioxidant, detoxification, lipid metabolism, and immunomodulatory pathways (ascorbate, ubiquinone, steroid…, biosynthesis); ↓ pro‐pathogen and inflammation‐linked pathways (quorum sensing, PTS…)	Not determined	Not determined	↓ TLR and cytokine gene expression	Zhen et al. 2021
Synbiotic (PoultryStar)	20 g/1000 chickens	1–60	Caecal	No information	↑ in Helicobacter, Lachnospiraceae, ↓ Ruminococcaceae, Clostridia; Both ↑ and ↓ Gastranaerophilales, Clostridia UCG‐014	Not determined	↑ BW of males, better survivability	↑ EW (30 wk), ↓ EW (40wk), ↑ shell weight, ↓ combined weight of yolk and albumen	Improved intestinal morphology (longer villi); Environment (house) effect also significant	(Prentza et al. 2022)
Sodium butyrate	1000 mg/kg	40–60	Caecal	↑ Chao1	↑ Lachnospiraceae, ↑ Ruminococcaceae	Not determined	↑ ELR, ↓ FCR	↑ hatching and chick weights, ↑ eggshell strength, ↓ eggshell colour	↓ serum lipid biomarkers; ↑ serum fat‐soluble vitamins, ↑ IgA, Improved intestinal morphology (↓ crypt depth, ↑ villi)	Xiao et al. 2023; Xu et al. 2023
*Enterococcus faecium*	6 × 10^8 CFU/kg	48–56	Caecal	No change	↓ Bacteroidetes	Not determined	No differences	↑ EW	↑ serum FSH levels	Wang et al. 2021a
Other functional additives										
Vitamin E	0.2 g/kg	66–75	Caecal	No change	↑ Lactobacillaceae, ↑ Veillonellaceae, ↑ Ruminococcaceae, ↑ Akkermansiaceae, ↑ Rikenellaceae	↑ DL‐Carnitine, ↑ Cuminaldehyde, ↑ Phylloquinone, ↑ Succinoylpyridine, ↑ 1‐Naphthol, ↓ acetylcholine	↑ ADFI	Not determined	(S)‐equol (+) corr. w/*Alistipes* and *Chlamydia*	Amevor et al. 2022
Metformin	75 mg/kg	25–65	Cloacal	No change	↑ Herbaspirillum, ↑ Cellulosilyticum, ↓ Acinetobacter	Not determined	Not determined	Not determined	Microbiome response is dose‐dependent (significant differences between doses)	Van Syoc et al. 2022

Abbreviations: ADFI, average daily feed intake, BW, bird weight, ELR, egg laying rate, EW, egg weight, FCR, feed conversion ratio.

Interventions such as quercetin, soyasaponins, grape seed extracts, herbal extracts, and quercetin‐vitamin E combinations have been tested in breeder hens. Although effects on α‐diversity are often limited, shifts in specific taxa are consistent: increases in Bifidobacteriaceae, Lactobacillaceae, Lachnospiraceae, Bacteroidetes, along with reduced prevalence of potentially harmful groups such as *Pseudomonas* (Proteobacteria) (Dittoe et al. 2019; Gao et al. 2025; Amevor et al. 2022; Grandhaye et al. 2020; Liu et al. 2023). These microbial changes coincide with metabolite shifts, including increases in DL‐carnitine, phylloquinone, and phenolic derivatives, which are linked to antioxidant capacity (Amevor et al. 2022). Functionally, these supplements have been associated with improved egg quality (e.g., greater shell strength, increased egg weight) and metabolic health, though some regimens (e.g., high‐dose short‐term grape seed extract) reduced body weight and feed conversion ratio (Grandhaye et al. 2020; Liu et al. 2023). Importantly, several studies highlight correlations between microbial taxa and anti‐aflatoxin activity or lipid metabolism, suggesting mechanisms beyond broad diversity changes (Amevor et al. 2022).

Butyrate‐focused strategies, including monobutyrin and *Clostridium butyricum* (CB), consistently improve gut barrier integrity, reduce inflammation, and increase serum protein levels (Feng et al. 2021; Xiao et al. 2023). While community‐level microbial shifts are modest, performance outcomes are clearer, with enhanced egg weight and reduced egg breakage. Synbiotics and yeast β‐glucan extend this probiotic‐focused approach: both have been associated with pathogen suppression, improved intestinal morphology, and greater microbial resilience (Zhen et al. 2021; Prentza et al. 2022). In addition, *Enterococcus faecium*, a commonly applied probiotic in poultry, has been linked to reductions in intestinal pathogens, stabilisation of gut microbial balance, and improvements in laying performance (Wang et al. 2021a). Together, these interventions act through enrichment of beneficial taxa and modulation of immune and antioxidant pathways, linking microbial composition more directly with reproductive outcomes.

Other functional/metabolic modulators, including vitamin E and metformin, also demonstrate potential to support microbial diversity and resilience (Amevor et al. 2022; Van Syoc et al. 2022). For instance, increased feed intake was observed due to supplementation of Vitamin E; However, their effects on the microbiota appear less direct and are often context dependent, varying with dose and basal diet.

Overall, evidence across categories shows that interventions rarely increase overall microbial diversity, but they consistently enrich specific beneficial groups (*Lactobacillus*, *Bifidobacterium*, Lachnospiraceae) and modify metabolite profiles. Performance improvements, including egg weight, shell strength, and feed efficiency, are more consistently documented than direct mechanistic links between microbiota shifts and reproductive outcomes. This suggests that while microbiome‐targeted nutrition is a promising strategy, more integrative studies coupling microbiota, metabolomics, and reproductive physiology are needed.

## 4 Breeder Hen Gut Microbiome and Its Link to Reproductive Performance

The breeder hen gut microbiome plays a central role in reproductive physiology, not only by influencing nutrient absorption and intestinal homoeostasis but also by directly interacting with the host endocrine and immune systems. Growing evidence indicates that variations in microbiota composition and function are associated with differences in egg laying rate (ELR), fertility, and hatchability (Shi et al. 2024; Dittoe et al. 2023). Importantly, these associations extend beyond simple correlations, implicating microbial metabolites and host–microbe signalling as key mediators of reproductive efficiency.

### 4.1 Hormone Metabolism and Endocrine Crosstalk

Reproduction in hens is tightly regulated by the hypothalamic–pituitary–ovary axis, which depends on coordinated secretion of gonadotropins and hormones such as follicle‐stimulating hormone (FSH), luteinising hormone (LH), and oestradiol (E2) (Du et al. 2020). Recent work has highlighted the gut microbiota as an important modulator of this system. For example, microbial β‐glucuronidase activity can deconjugate oestrogens in the gut, thereby increasing their enterohepatic recirculation (Hu et al. 2023). This microbial modulation of circulating hormone pools suggests a route through which gut dysbiosis may impair ovulation or follicular maturation.

Correlative evidence supports this link where a study found that the genus *Hungatella* correlated strongly with serum E2 levels during different egg‐laying stages, reinforcing the idea that microbial taxa can influence steroid biosynthesis (Shi et al. 2024). Additionally, *Clostridium sensu stricto, Bifidobacterium* and *Rhodococcus* have been positively correlated with E2, FSH and LH (Gao et al. 2025). Intervention studies further strengthen causality. For example, Cao et al. (2023) demonstrated that faecal microbiota transplantation (F from high‐performing donors increased serum E2 and upregulated ovarian receptor genes (ESR1, ESR2, FSHR, AMHR) in low‐performing hens (Cao et al. 2023). This was associated with improved egg production and ovarian development, providing functional proof that microbial modulation can alter reproductive endocrinology. It was similarly reported that microbiota transfer enhanced egg production, suggesting that targeted microbial therapies may be viable tools for improving breeder productivity (Wang et al. 2021b).

### 4.2 Intestinal Health, Nutrient Utilisation, and Energy Partitioning

Efficient reproduction is energetically demanding, requiring optimal nutrient digestion and absorption. The gut microbiome contributes to these processes both structurally and metabolically. Structurally, beneficial microbes enhance villus height, reduce crypt depth, and strengthen tight junction integrity, all of which increase absorptive capacity (Amevor et al. 2022). Metabolically, microbial fermentation of dietary fibre produces short chain fatty acids (SCFA) such as acetate, propionate, and butyrate, which serve as energy substrates for enterocytes and modulators of gut homoeostasis (Pangga et al. 2025).

The functional significance of SCFAs extends beyond intestinal energy balance. SCFAs act as signalling molecules, regulating hormones like leptin (Acharya et al. 2024) and glucagon‐like‐peptide 1 (GLP‐1) via multiple pathways, which influence lipid metabolism and reproductive energy allocation (Zhang et al. 2019). Propionate, in particular, support ovarian function of sows by reducing oxidative stress‐induced apoptosis of granulosa cells, thereby promoting follicular development (Qin et al. 2025). In poultry, supplementation with SCFAs has improved reproductive performance, egg quality, and shell thickness in aged hens (Sengor et al. 2007), underlining their practical relevance. Moreover, Shi et al. (2024) identified metabolites such as 3‐phenyllactic acid, quinic acid, and caffeic acid that correlate with high egg production, suggesting that microbial metabolomics may provide biomarkers for reproductive efficiency (Shi et al. 2024).

### 4.3 Immunoregulation and Inflammation

Intestinal inflammation is increasingly recognised as a reproductive burden, reducing egg production and impairing hatchability (Nii et al. 2020; Zhai et al. 2022). For example, exposure to deoxynivalenol induces pro‐inflammatory responses that damage intestinal villi and disrupts tight junction integrity, leading to dysbiosis characterised by a loss of beneficial genera like *Lactobacillus* and expansion of pathogenic taxa such as *Proteobacteria* and *Spirochaetes*. This imbalance compromises nutrient absorption and systemic immune balance, ultimately lowering laying performance (Zhai et al. 2022). More broadly, dysbiosis can promote overgrowth of pathobionts, chronic inflammation, and reduced metabolic activity, which deprives the host of key microbial products needed for energy and reproductive function (Kogut 2019). Thus, intestinal inflammation not only induces local gut damage but also undermines the microbial–immune homoeostasis required for optimal egg production and fertility in hens. In fact, FMT from high‐laying donors has been shown to reduce pro‐inflammatory cytokines while improving ovarian function (Cao et al. 2023), providing mechanistic evidence that microbiota support reproductive longevity.

### 4.4 Microbial Taxa, Diversity and Contradictions

Specific bacterial taxa are recurrently associated with reproductive performance. *Butyricicoccus, Enterococcus, Bacteroides, Bifidobacterium*, and *Lactobacillus* are generally correlated with higher ELR, improved intestinal function, and reduced ovarian apoptosis (Gao et al. 2025; Yang et al. 2020; Cao et al. 2023). Similarly, *Bacillus, Rhodanobacter*, and *Streptomyces* have been linked with average ELR, and ovarian gene expression, such as the BMPR1B gene (Wang et al. 2021a, 2021b). However, the role of *Lactobacillus* is particularly controversial. While many studies report positive correlations, one study found *Lactobacillus* dominance in poor feed converters, suggesting that its effects are strain‐ and context‐dependent (Díaz‐Sánchez et al. 2019).

Likewise, the relative contributions of Firmicutes and Proteobacteria vary: Firmicutes enrichment has been linked with high ELR (Yang et al. 2020; Cao et al. 2023), while J. Wang et al. (2021a) associated higher Proteobacteria (particularly Gammaproteobacteria) with average ELR breeders (Wang et al. 2021a). Microbial diversity has also yielded contradictory results. Yang et al. (2020) (Yang et al. 2020) indicated that higher diversity in the small intestines (duodenum and ileum) might negatively impact reproduction, whilst other authors both observed that higher alpha‐diversity (including observed species, Chao1, ACE, and Shannon indices) in the caecum was associated with better egg‐laying performance (Wang et al. 2021a; Cao et al. 2023). Such discrepancies may reflect gut‐region specificity (ileum vs caecum), host genetic background, or methodological differences in sequencing and analysis.

### 4.5 Integrating Metabolomics With Microbiome Data

A key challenge in this field is moving beyond taxonomic descriptions toward functional understanding. Metabolomic profiling offers a promising solution by linking specific microbial taxa to biochemical pathways. For instance, *Megasphaera* was positively associated with amino acid metabolism and metabolites such as quinic acid and folic acid, while *Hungatella* correlated with E2 biosynthesis (Shi et al. 2024). Integrative approaches can help explain why the same taxa may have variable effects in different contexts, providing a mechanistic framework for predicting breeder performance.

## 5 Gut Microbial Transfer From Breeder to Progeny

**Figure 3:**
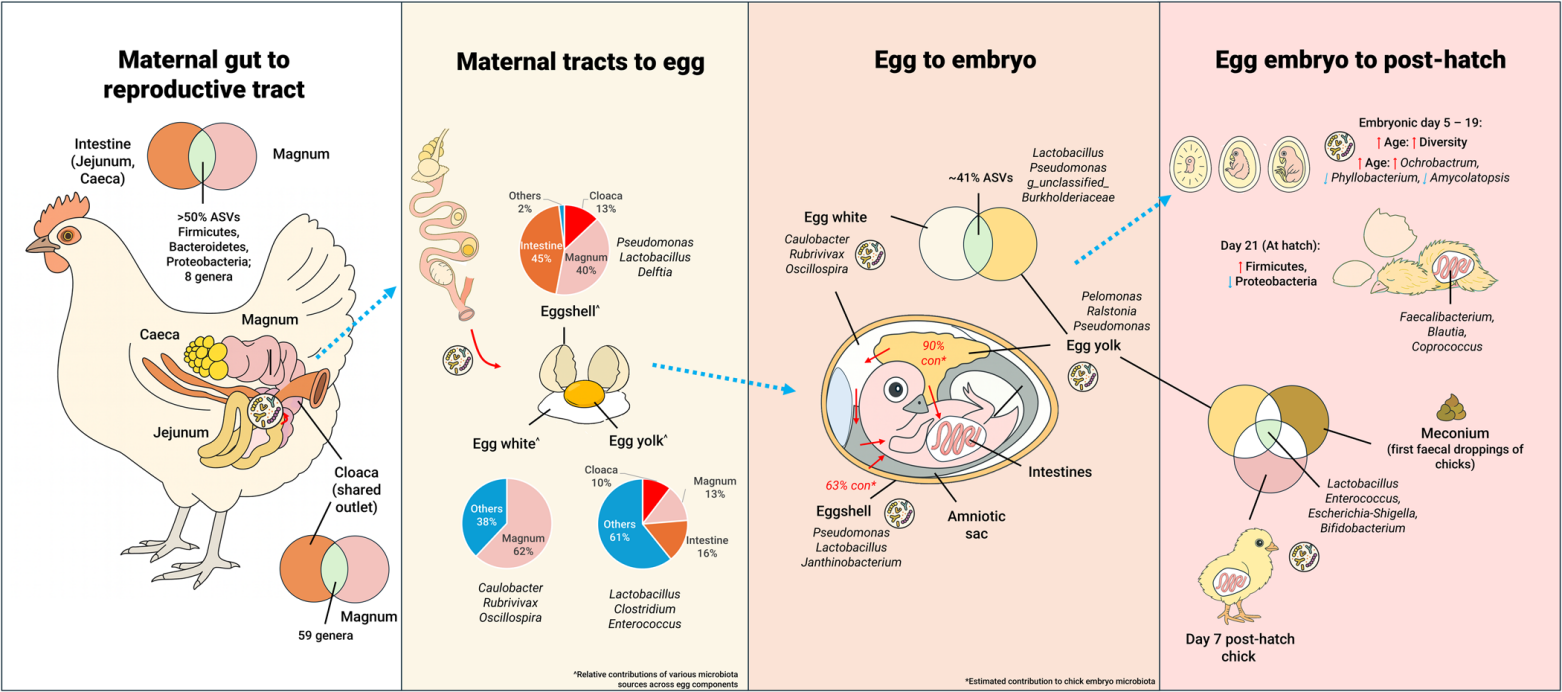
Overview of microbial transfer from breeder hen to progeny.

There is compelling evidence for vertical microbial transmission from the mother hen to the chick, influencing early gut colonisation. This vertical transmission plays a critical biological role in the offspring's metabolism, nutrition, physiology, and immunoregulation, and is crucial for the early establishment of robust microbial communities, influencing long‐term health and performance. Importantly, recent work has demonstrated that this process is not uniform but genotype‐dependent: core vertically transmitted taxa such as *Bifidobacterium* and *Lactobacillus* are regulated by heritable host variants, with overlapping genomic regions identified in both hens and their chicks. These host‐microbe interactions are enriched in pathways linked to immune development and metabolic regulation, indicating that the genetic background of the breeder partly determines which microbes are reliably inherited across generations (Gao et al. 2025). A summary of the breeder‐to‐progeny microbial transfer overview is shown in Figure 3.

### 5.1 Maternal Gut and Reproductive Tract

Microbial transfer begins with movement from the maternal GIT to the reproductive tract (Lee et al. 2019; Shterzer et al. 2020). The cloaca, a shared outlet for the digestive and reproductive systems, provides a direct route for such exchange (Shterzer et al. 2020). This physiological connection results in a striking overlap: 55% of ASVs in the jejunum and 53% in the caecum were also detected in the magnum, a central region of the oviduct. At the individual level, approximately 83.6% of jejunal reads and 52.1% of caecal reads overlapped with those in the magnum, and the likelihood of a microorganism appearing in the oviduct was directly proportional to its relative abundance in the gut (Shterzer et al. 2020). The cloaca shares 31 genera with the small intestine and 59 with the magnum, with 110 shared genera between all compartments (Gao et al. 2025). This shared microbial pool can be further expanded through soyasaponin (SS) supplementation, which notably increases genera like *Rhodococcus* and *Pseudomonas* (Gao et al. 2025). Both digestive and reproductive systems are dominated by *Firmicutes, Bacteroidetes*, and *Proteobacteria* (Lee et al. 2019; Shterzer et al. 2020), although the oviduct favours aerobic taxa such as *Burkholderiales, Pseudomonadales*, and *Bacillales*, reflecting its oxygen‐rich niche. Importantly, the oviduct microbiota appears stable along its length, with no significant differences among the infundibulum, magnum, isthmus, and shell gland (Shterzer et al. 2020).

### 5.2 Maternal Transfer to Egg

Microbiota present in the maternal oviduct and cloaca can be vertically transmitted to the developing egg (Lee et al. 2019; Gao et al. 2025). Source‐tracking analyses estimate that 17% of eggshell microbiota originates from the cloaca and 28% from the magnum (Lee et al. 2019). (Gao et al. 2025) further corroborated this, finding that a significant portion of the eggshell microbiota originates from the cloaca (~13%) and magnum (~40%), and a considerable part also derives from the maternal intestinal microbiota (~45%), thus highlighting the multiple maternal reservoirs that seed the egg surface.

Within the egg, the yolk sac plays a particularly important role as both a nutrient reservoir and a conduit for microbial transfer (Gao et al. 2025; Wong and Uni 2021). Microbial community analyses also show that the yolk sac (embryonic day 19) microbiota resembles that of the maternal intestine (15.5%), magnum (13.3%), and cloaca (10.4%), suggesting contributions from multiple maternal sites (Gao et al. 2025). Among the core taxa consistently detected across these compartments are *Lactobacillus, Clostridium*, and *Enterococcus* (Gao et al. 2025).

The magnum also directly contributes to vertical transfer, seeding both egg white and embryonic gut. Source‐tracking analysis revealed that 61.89% of microbes in the egg white, and 20.95% and 72.1% in the embryonic guts at 15 and 21 days, respectively, originated from the magnum (Gong et al. 2023). Dominant genera simultaneously present in the magnum mucosa, egg white, and embryonic gut include *Caulobacter*, *Rubrivivax*, *Oscillospira*, *Methylibum*, *Pseudomonas*, and *Erwinia (*Gong et al. 2023 *)*.

Past evidence also supports vertical transmission of pathogens from hen to egg. *Salmonella Enteritidis* has been shown to colonise the ovary, infundibulum, and magnum, leading to deposition into yolk membranes, albumen, and shell membranes during egg formation (Gantois et al. 2009). *Campylobacter* can also be passed via both ovarian and cloacal routes, with the organism detected in fertile egg contents, hatchery fluff, yolk material, and embryonic intestines (Cox et al. 2012). In contrast, more recent work suggests that under commercial conditions, vertical transmission is relatively inefficient, since most maternal ASVs are absent from chicks; nevertheless, certain taxa, such as *Lactobacillus* and members of the *Enterobacteriaceae*, appear to be reliably transmitted (Shterzer et al. 2023).

### 5.3 Egg to Chick Embryo

As established above, both the egg yolk and egg white carry substantial microbiota of maternal origin, which are subsequently transferred to the developing embryo. Gong et al. (2023) (Gong et al. 2023) proposed that microbes present in the egg white may pass the amniotic membrane and subsequently colonise the embryonic gut after being ingested by the developing embryo. Indeed, microbial communities of the egg white and the embryonic gut were reported not to be significantly different in another study (Lee et al. 2019). The eggshell microbiota has also been attributed as a source of microbiota for the embryo. Approximately a third of the microbiota (63%) of the chick embryo at embryonic day 18 has been sourced from eggshells (Lee et al. 2019).

Further evidence highlights the yolk as a dominant source of microbiota. Specifically, 89.83% of the intestinal microbiota of chicken embryos at embryonic day 19 was estimated to originate from the yolk (Ding et al. 2021). Shared genera between these two compartments included *Pelomonas* (45% and 49%, respectively) and *Ralstonia* (12% and 11%, respectively). Interestingly, a decreasing abundance of *Proteobacteria* was observed from embryonic day 7 to 15, but increased at day 19 (Ding et al. 2021). Complementary evidence from (Gao et al. 2025) reinforced this finding, identifying the yolk sac as a critical conduit for vertical microbial transmission. Although the yolk generally harbours a higher microbial abundance than the egg white (Jin et al. 2022), the egg white is far from sterile. Proteobacteria, Firmicutes, Actinobacteria, and Bacteroidetes were detected in both yolk and egg white, with microbial overlap between compartments. Specifically, a 20% ASV overlap was reported between egg yolks and whites of fresh eggs, whereas a 41% overlap was observed in incubated eggs at day 12 (Jin et al. 2022).

### 5.4 Embryo to Post‐Hatch

As incubation progresses, the embryonic gut microbiota undergoes dynamic restructuring. More than 20 phyla and over 600 genera could be detected across embryonic development, while diversity and composition shift markedly between stages (Akinyemi et al. 2020). Core taxa such as *Ochrobactrum, Phyllobacterium*, and *Amycolatopsis* were present at all stages, but with changing abundances: *Phyllobacterium* increased substantially toward day 19, whereas *Ochrobactrum* and *Amycolatopsis* declined (Akinyemi et al. 2020). By embryonic day 19, microbial diversity was significantly greater than at earlier stages, accompanied by predicted enrichment of pathways involved in energy metabolism and immune development, underscoring the role of the microbiota in preparing the chick for post‐hatch life (Akinyemi et al. 2020).

The transition from embryo to hatchling is accompanied by further microbial reshaping as residual yolk contents are absorbed into the intestine. Further, a previous study (Ding et al. 2021) demonstrated that yolk microbes directly enter the intestine in late embryogenesis and even persist for several days after hatch, seeding the neonatal gut. This early community, dominated by *Proteobacteria, Firmicutes*, and *Bacteroidetes*, is subsequently enriched with taxa such as *Faecalibacterium, Blautia, Coprococcus, Dorea*, and *Roseburia*, which are associated with SCFA production and intestinal development. It was further revealed that maternal oviductal, cloacal, and intestinal microbes are shared not only with the yolk sac and meconium but also with the early chick caecum, with *Lactobacillus, Enterococcus, Escherichia‐Shigella*, and *Bifidobacterium* among the reliably transmitted genera (Gao et al. 2025). Importantly, differences in yolk‐associated microbiota have also been linked to hatching success, with higher hatchability associated with the presence of *Muribaculaceae* in yolks after 12 days of incubation, whereas greater abundance of *Rothia* in fresh yolks has been correlated with reduced hatchability (Jin et al. 2022).

External and temporal factors surrounding incubation also shape hatchability and early microbial establishment. Prolonged litter exposure has been shown to elevate microbial load on the eggshell and, after 16 h, significantly reduce hatchability through increased embryonic mortality, with further variation linked to breeder flock age (Csitári et al. 2025). Meanwhile, the timing of hatch itself can influence early gut colonisation: late‐hatched chicks exhibited distinct caecal profiles characterised by reduced Firmicutes and higher Bacteroidetes abundances at day 11, differences that were accompanied by altered growth trajectories (Such et al. 2023).

## 6 Impact of Maternal Interventions on Progeny Microbiome and Performance

Interventions at the breeder level have shown a significant impact on the microbiome of their progeny, suggesting that maternal nutrition plays a crucial role in shaping the early gut microbial communities of offspring chickens (Gao et al. 2025). Various studies have investigated the effects of various supplements administered to breeder hens on the intestinal health and microbial composition of their chicks.

One key intervention involves maternal SS supplementation, which has been shown to facilitate the colonisation of *Bifidobacterium* in both maternal and offspring microbiota, contributing to microbial inheritance (Gao et al. 2025). This led to a significant increase in the quantity of *Bifidobacterium adolescentis* in the gut microbiome of 7‐day‐old chicks, and SS interacts with this bacterium to produce γ‐aminobutyric acid, which modulates offspring intestinal development. SS notably altered the shared bacterial genera between mothers and offspring and within different embryonic compartments, impacting the vertical transmission of maternal microbiota. Similarly, maternal genistein supplementation improved the gut microbiota of offspring by enhancing the abundance of *Escherichia coli* and reducing Gammaproteobacteria in newly hatched chicks (Gao et al. 2023). This intervention also mitigated the risk of bacterial diversity impairment in offspring challenged with lipopolysaccharide (LPS). Specifically, at 1 day of age, unidentified *Clostridiales* were reduced, while unidentified *Enterobacteriaceae* and *Epulopiscium* increased in the meconium. In LPS‐challenged offspring at 21 days, maternal genistein resulted in a higher relative abundance of beneficial bacteria such as *Lactobacillus* and *Barnesiella*. Also, maternal supplementation with CB significantly elevates the growth performance of offspring, leading to increases in final bird weight and average daily gain, and decreased feed:gain, with high‐level CB showing more pronounced effects (Wang et al. 2021b).

Further interventions include maternal stevioside supplementation, which effectively prevented the impairment of bacterial diversity induced by LPS challenge in chicken offspring (Jiang et al. 2021). Offspring from stevioside‐supplemented hens exhibited a significantly increased abundance of *Lactobacillus* and enriched pathways related to bacterial adhesion and colonisation (Jiang et al. 2021). Lastly, dietary supplementation of laying breeder hens with a combination of β‐carotene, curcumin, allicin, and sodium butyrate resulted in significant enrichment of *Firmicutes* in the jejunum of their offspring chicks, including a slightly higher presence of *Enterococcus* and a lower *Lactobacillus* at the genus level, which was suggested to contribute to enhanced jejunal immunity (Gong et al. 2020). The same authors suggested the improved immunity of offspring is due to the transfer of microbes from the maternal magnum to the egg white, as a consequence of improved nutrition of the breeder hen (Gong et al. 2023). These diverse strategies underscore the potential for breeder‐level dietary modifications to programme offspring gut health and immune development through vertical microbial transmission.

## 7 Future Direction and Conclusion

**Figure 4:**
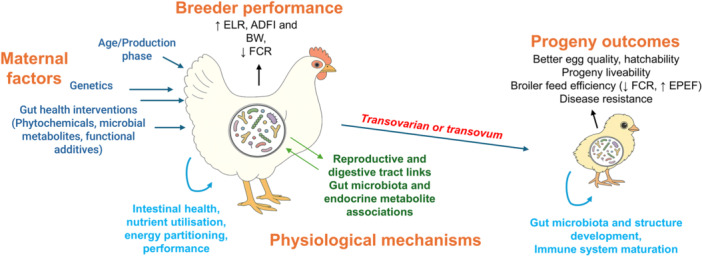
Overview of microbial legacy from breeder hen to progeny.

This review underscores the breeder hen gut microbiome as a dynamic and multifactorial system shaped by age, genetics, and dietary interventions, with far‐reaching implications for maternal health, reproductive performance, and progeny development (Figure 4). Strong evidence supports vertical microbial transmission through the cloaca and egg components, establishing a “microbial legacy” that seeds the offspring's gut and influences immune maturation, intestinal development, and growth trajectories.

Despite progress, key gaps remain. The majority of current research relies on 16S rRNA amplicon sequencing, which limits taxonomic resolution and often obscures functionally relevant differences at the species or strain level. Given that beneficial and pathogenic strains can coexist within the same genus, future studies must incorporate higher‐resolution approaches such as shotgun metagenomics, strain‐level profiling, and culture‐based validation to more accurately identify microbial contributors to performance and vertical transmission. Multi‐omics strategies, including metabolomics, transcriptomics, and proteomics, are also essential to link microbial shifts with functional outputs, particularly in elucidating how microbial metabolites regulate ovarian physiology, embryonic development, and immune programming.

Additional research should focus on the mechanisms governing microbial selection and survival across reproductive tract niches and during embryo incubation, where the antimicrobial properties of the egg white pose unique selective pressures. Disentangling the relative contributions of vertical versus environmental acquisition is also crucial, especially given the variability of hatchery and incubation conditions that influence microbial colonisation and hatchability. Furthermore, contradictory findings regarding the roles of Firmicutes and Proteobacteria in reproductive performance highlight the importance of integrating host genetics, gut segment specificity, and microbial network interactions into study designs.

In conclusion, the breeder hen gut microbiome represents a dynamic ecosystem and a powerful form of nongenetic inheritance. By strategically modulating maternal microbiota through diet and management, it is possible to enhance egg quality, hatchability, and progeny performance. Realising this potential, however, requires a shift toward strain‐resolved, multi‐omics research coupled with standardised, longitudinal, and mechanistic studies. Such approaches will be key to unlocking the full scope of maternal microbial programming, offering innovative avenues to improve poultry health, productivity, and sustainability.

## Author Contributions

**Gladys Maria Pangga:** conceptualization, methodology, validation, data curation, formal analysis, investigation, visualization, writing – original draft. **Anne Richmond:** conceptualization, supervision, funding acquisition, writing – review and editing. **Ozan Gundogdu:** conceptualization, validation, investigation, resources, supervision, project administration, funding acquisition, writing – original draft, writing – review and editing. **Stephen Bamford:** conceptualization, writing – review and editing. **Umer Zeeshan Ijaz:** validation, data curation, formal analysis, investigation, supervision, writing – review and editing. **Nicolae Corcionivoschi:** writing – review and editing.

## Ethics Statement

The authors have nothing to report.

## Consent

The authors have nothing to report.

## Conflicts of Interest

A.R. and S.B. are employed by the company Pilgrim's Europe Ltd (Moy Park). The other authors declare no conflicts of interest.
